# Development and Initial Analytical Evaluation of a Multiplex PCR-Based Targeted NGS Assay for Expanded Viral Detection in Clinical Respiratory Specimens

**DOI:** 10.3390/diagnostics16182888

**Published:** 2026-09-08

**Authors:** Maksim I. Nadtoka, Anna Y. Bukharina, German V. Roev, Arina V. Peresadina, Anastasiia V. Vykhodtseva, Matvei R. Agletdinov, Sergey E. Goncharov, Ekaterina V. Pimkina, Elmira R. Samitova, Margarita D. Khaldeeva, Ruslan F. Sayfullin, Yana A. Voytsekhovskaya, Anna S. Cherkashina, Alexander E. Kuznetsov, Kamil F. Khafizov, Vasiliy G. Akimkin

**Affiliations:** 1 Central Research Institute of Epidemiology, Moscow 111123, Russia; nadtoka@cmd.su (M.I.N.); annabukharina8@gmail.com (A.Y.B.); roevherman@gmail.com (G.V.R.); peresadina@cmd.su (A.V.P.); agletdinov@cmd.su (M.R.A.); vgakimkin@yandex.ru (V.G.A.); 2 Department of Biotechnology, D. Mendeleyev University of Chemical Technology of Russia, Moscow 125047, Russia; 3 Department of Pediatrics and Pediatric Infectious Diseases, First Moscow State Medical University (Sechenov University), Moscow 119048, Russia; 4 Children’s Hospital of Z.A. Bashlyaeva, Moscow 125373, Russia; 5 Department of Infectious Diseases in Children, Pirogov Russian National Research Medical University, Moscow 117513, Russia; 6 Federal Research and Clinical Center of Intensive Care Medicine and Rehabilitology, Moscow 107031, Russia

**Keywords:** respiratory viruses, infections, tNGS, multiplex PCR, molecular diagnostics, bioinformatics

## Abstract

**Background:** The diagnosis of infectious diseases currently relies predominantly on polymerase chain reaction (PCR) assays. However, the range of pathogens that can be detected simultaneously using these methods is limited, and their effective use depends on an a priori etiological hypothesis. Multiplex PCR-based targeted next-generation sequencing (mp-tNGS) is considered a promising complementary approach for expanded viral testing. This study aimed to develop an in-house mp-tNGS method, perform an initial evaluation of its analytical sensitivity, and explore its performance using clinical respiratory specimens. **Methods**: The method comprised a panel targeting 28 viruses and bioinformatics pipelines for mp-tNGS data processing. Analytical sensitivity was evaluated using positive control samples (PCSs) containing encapsulated RNA or DNA. Performance with clinical specimens was explored using 910 nasopharyngeal and oropharyngeal swab specimens, which were divided into three groups based on the results of prior PCR testing. **Results**: Of the 28 viral targets, 23 underwent experimental analytical evaluation, whereas five were assessed in silico only. For 19 of the 23 PCSs tested, the estimated limit of detection (LoD) ranged from 6.3 × 10^2^ to 9.4 × 10^3^ copies/mL. In the group of 216 PCR-positive specimens, PCR and mp-tNGS results were fully concordant in 139 cases; in 28 specimens, mp-tNGS additionally detected other viruses. Among 394 PCR-negative specimens, mp-tNGS detected viruses in 81. In the group of 300 hospital-derived specimens, mp-tNGS detected at least one viral target in 131 specimens, including additional viral detections and coinfections not identified during the initial testing. A substantial proportion of the additional findings involved Epstein–Barr virus (EBV) and cytomegalovirus (CMV), warranting cautious clinical interpretation. **Conclusions**: The developed mp-tNGS approach demonstrated the potential to simultaneously detect a broad range of known viruses, provide viral typing, and identify coinfections. The method may be used as an adjunctive tool to broaden the laboratory diagnosis of respiratory infections; however, further optimization, validation, and standardization of criteria for result interpretation are required.

## 1. Introduction

Acute respiratory infections (ARIs) remain a major public health concern, with approximately 80% of cases attributed to viral etiology [1]. The COVID-19 pandemic, caused by SARS-CoV-2, demonstrated how rapidly spreading respiratory viruses can substantially alter the epidemiological landscape and have a significant socioeconomic impact [2]. However, the range of viruses included in routine diagnostic algorithms varies considerably among laboratories and is often limited to the most common pathogens, including influenza A and B viruses (FluA/B), severe acute respiratory syndrome coronavirus 2 (SARS-CoV-2), human respiratory syncytial virus (HRSV), and, in some cases, seasonal human coronaviruses [1]. Other respiratory viruses, such as parainfluenza viruses, human metapneumovirus, adenoviruses, parechoviruses, and bocaviruses, are much less frequently covered by diagnostic panels, despite their substantial contribution to the overall burden of respiratory infections [3,4,5,6,7,8,9,10]. Some of these viruses can also cause localized outbreaks in institutional settings, making their rapid identification important for the timely implementation of preventive measures [6].

Members of the family *Herpesviridae* warrant separate consideration. Although varicella-zoster virus (VZV), cytomegalovirus (CMV), and Epstein–Barr virus (EBV) are not typically considered causative agents of acute respiratory viral infections, their detection in respiratory specimens may be clinically relevant in neonates, pregnant women, immunocompromised patients, and transplant recipients. VZV can cause severe disease in adults and individuals with immunodeficiency [11], while CMV is associated with the risk of congenital infection and severe disease in immunocompromised patients [12]. EBV is associated with infectious mononucleosis, lymphoproliferative disorders, and malignancies [13]. Herpesviruses may also reactivate during acute respiratory viral infections and contribute to disease progression, particularly in immunocompromised patients [14,15]. Their inclusion in expanded diagnostic panels may therefore be justified. However, their detection should be interpreted only in the appropriate clinical context.

The increasing number of viruses posing a threat to human health, together with the diversity of their transmission settings, highlights the limitations of conventional diagnostic methods. This underscores the need for novel approaches capable of detecting a broader range of pathogens. Although polymerase chain reaction (PCR) remains the gold standard and a primary diagnostic method, its multiplexing capacity is generally limited to approximately five targets per reaction [16,17]. Consequently, multiple PCR assay kits are often required to cover a broader range of targets, with each assay involving several separate reaction mixtures. However, their rational and efficient use depends on knowledge of the likely causative agent. These limitations may reduce the likelihood of detecting certain pathogens and complicate the identification of coinfections [1,18,19].

In this context, next-generation sequencing (NGS) technologies represent a promising alternative approach for the diagnosis of infectious diseases. NGS technologies have evolved beyond their initial use as research tools and are increasingly being implemented in clinical laboratories, driven by rapid technological advances and a substantial reduction in sequencing costs [20]. These technologies have significantly broadened the scope of pathogen detection, allowing for the identification of a wide range of causative agents without prior pathogen selection or the use of pathogen-specific primers or probes [1,20,21]. This approach enables the identification of both established and previously uncharacterized pathogens and is known as metagenomic next-generation sequencing (mNGS) [22]. Nowadays it is frequently used for the comprehensive detection of bacteria, viruses, fungi, and parasites directly in clinical specimens [22]. Despite its broad applicability, mNGS often exhibits lower sensitivity than targeted methods, largely due to the high proportion of host-derived nucleic acid sequences in metagenomic datasets [21]. In addition, mNGS remains relatively costly and often requires additional steps for selective depletion of host DNA and RNA [23], substantial computational resources for data analysis, and high-throughput sequencing platforms.

Although mNGS is the most broadly utilized approach for determining the etiology of infections caused by previously uncharacterized pathogens or when no a priori etiological hypothesis is available, targeted next-generation sequencing (tNGS) represents a promising alternative for the diagnosis of infections caused by known pathogens. Targeted NGS involves the prior enrichment of pathogen nucleic acid sequences using specific primers or probes, thereby selectively increasing their abundance in the sequencing library. As a result, a larger proportion of sequencing reads is assigned to the target pathogens, enabling the analysis of a greater number of specimens while reducing the cost per specimen [23,24]. Additionally, tNGS generally provides higher analytical sensitivity than mNGS and, despite its predefined target range, can cover a sufficiently broad spectrum of pathogens [23,24].

A promising approach for the diagnosis of infectious diseases is multiplex PCR-based targeted next-generation sequencing (mp-tNGS), which employs multiplexed primer panels for the simultaneous amplification of multiple pathogen targets. This approach enables the detection of dozens or even hundreds of pathogens in a single analysis, while the overall sample-to-result workflow can be completed within approximately 10–24 h, depending on the protocol and sequencing platform used [25,26]. Thus, mp-tNGS offers a compromise between turnaround time and the breadth of pathogen coverage.

The aim of this study was to develop a multiplex PCR-based tNGS panel for the detection and subtyping of 28 viral targets, most of which are associated with respiratory tract infections, and to evaluate its analytical performance and applicability to clinical respiratory specimens. The study was designed to characterize the initial analytical performance of the developed panel, assess its concordance with prior PCR findings, and evaluate the additional detection yield associated with broader target coverage in archived clinical respiratory specimens. Experimental analytical evaluation was completed for 23 targets, whereas five targets could be assessed in silico only because suitable positive materials were unavailable. mp-tNGS may serve as an expanded approach for detecting known respiratory viruses and coinfections, particularly when standard PCR assays do not cover the full range of potential causative agents.

## 2. Materials and Methods

### 2.1. Selection of Viral Targets

To assess the feasibility of simultaneously identifying viral pathogens associated with acute respiratory infections (ARIs), we developed a multiplex panel for the targeted sequencing of short conserved regions within the genomes of 28 viral pathogens or their subtypes. The complete list of targets included in the panel design is presented in Table 1. The composition of a targeted sequencing panel should reflect the local epidemiological context, including pathogen prevalence and seasonality, the characteristics of the tested population, and current surveillance priorities. The present panel was designed to expand the range of viruses detectable in respiratory specimens in the local clinical setting and to provide type- or subtype-level information for selected epidemiologically important viruses.

It should be noted that human rhinoviruses (HRVs), despite their substantial contribution to the burden of acute respiratory viral infections, were not included in the panel. This decision was based on the high genetic variability of HRVs and the existence of more than 160 genotypes within species A, B, and C. The extensive genetic diversity of HRVs complicates the selection of conserved primer-binding sites capable of detecting the full range of viral genotypes. However, as part of the further improvement of our mp-tNGS approach, a panel module for HRV detection is currently being developed. In contrast, the inclusion of herpesviruses in the panel is not intended for the diagnosis of typical acute respiratory viral infections but solely for the detection of potentially clinically significant reactivations or coinfections in specific patient groups.

### 2.2. Primer Design

Designing a primer panel containing a large number of primers presents several technical challenges. First, primers combined into one or two reaction mixtures must provide sufficient sensitivity and specificity, have an optimal GC content and similar annealing temperatures, and exhibit minimal primer-dimer formation. In addition, the target amplicons must provide sufficient sequence information for viral typing. In our case, primer selection was further complicated by the addition of a Nextera adapter to the 5′ end of each target-specific oligonucleotide, which makes some degree of primer-dimer formation virtually unavoidable. At the same time, the presence of this adapter reduces the number of sample-handling steps required during sequencing library preparation and thereby lowers the risk of cross-contamination. It should also be noted that extensive primer-dimer formation during multiplex PCR is partly attributable to the absence of cognate templates for the vast majority of primers in the reaction mixture.

Full-length or partial genomic sequences of the target viruses retrieved from the National Center for Biotechnology Information (NCBI) GenBank database were used for primer selection. For each target, multiple sequence alignment was performed to identify conserved regions suitable for detection and, where specified by the panel design, differentiation of individual viral types or subtypes. Primers were selected using Primer-BLAST, taking into account melting temperature, GC content, and amplicon length. Primer specificity was evaluated in silico by comparison with sequences available in NCBI databases. The final primer pairs generated amplicons ranging from 117 to 395 bp, excluding adapter sequences.

Singleplex experimental evaluation was performed for 23 of the 28 targets. Each primer pair was tested by singleplex PCR using positive specimens obtained from a clinical laboratory; the corresponding target virus had previously been detected in these specimens using a registered in vitro diagnostic PCR assay. Experimental validation using positive biological specimens was not performed for HPIV-4b, HBoV-2, HBoV-3, HPeV-1 or HPeV-3 due to the absence of suitable positive specimens. Thus these targets were evaluated in silico only. The resulting amplicons were visualized by gel electrophoresis and assessed for the expected amplicon size, the absence of nonspecific amplification products, and the absence of primer dimers. The amplicons were subsequently sequenced on an Illumina platform to assess the specificity of the resulting sequences. The experimentally validated primer pairs and the five pairs evaluated only in silico were then combined into two pools, taking into account both predicted and experimentally observed interactions among the primers.

### 2.3. Clinical Specimens

A total of 910 nasopharyngeal and oropharyngeal swab specimens were analyzed in this study and divided into three main groups. The clinical specimens were received by the laboratory between January 2024 and March 2025.

Group I (*n* = 216) comprised positive specimens with a previously identified viral pathogen. These specimens were obtained from the CMD clinics in Moscow following routine laboratory PCR testing using the following registered assays: ORVI-Screen-FL and/or Influenza Virus A/B-FL (AmpliSens^®^).

Group II (*n* = 394) comprised specimens that had tested negative during prior laboratory PCR testing using the ORVI-Screen-FL or COVID-19-FL assay (AmpliSens^®^, Moscow, Russia). These specimens were also obtained from the CMD clinics in Moscow.

Group III (*n* = 300) comprised specimens, some of which had tested positive for a viral infection during prior PCR testing using registered assays. The specimens were collected from patients presenting with signs of acute respiratory viral infection at the Children’s Hospital of Z.A. Bashlyaeva in Moscow.

Across all three groups, specimens that tested positive exclusively for human rhinoviruses (HRVs) by PCR were excluded because the panel did not include primers targeting these viruses. The study, which included retrospective and prospective components, was approved by the Local Ethics Committee of the Central Research Institute of Epidemiology of the Federal Service for the Oversight of Consumer Protection and Welfare (Protocol No. 147, dated 17 December 2024). Specimens obtained before 17 December 2024, were residual clinical specimens originally collected during routine diagnostic testing. At the time of collection, patients or their legal representatives provided informed consent permitting the subsequent use of residual biological material for scientific research. No procedures specific to the present study, including specimen selection or mp-tNGS analysis, were initiated before approval was granted by the Local Ethics Committee. The approved protocol covered the analysis of these archived specimens. Specimens collected after that date were prospectively included in the study after informed consent had been obtained from the patients or their legal representatives. Specimen collection and analysis were performed in accordance with applicable ethical standards.

### 2.4. Nucleic Acid Extraction and Reverse Transcription

Total nucleic acids (RNA and DNA) were extracted using the RIBO-prep reagent kit (AmpliSens^®^, Russia) according to the manufacturer’s instructions. Nucleic acids were eluted in 25 µL of the buffer supplied with the kit. Reverse transcription was performed using the REVERTA-L kit (AmpliSens^®^, Russia). Unlike the standard protocol, the resulting complementary DNA (cDNA) was not diluted in the DNA buffer supplied with the kit. The resulting cDNA/DNA mixture was subsequently used as the template for multiplex PCR.

### 2.5. Multiplex PCR

Target regions of the viral genomes were amplified in two parallel multiplex PCR reactions using the developed primer panel (Supplementary Table S1). The oligonucleotides included in the panel were divided into two mixtures (pools) to reduce primer-dimer formation during PCR. The composition of the pools is provided in Supplementary Tables S2a,b.

Pool 1 contained primer pairs targeting HPeV-1, HPeV-3, HPIV-1, HPIV-2, HPIV-3, HPIV-4a, HPIV-4b, HMPV-A, HMPV-B, HBoV-1, HBoV-2, HBoV-3, HRSV-A and HRSV-B (Supplementary Table S2a). Pool 2 contained primer pairs targeting FluB/Victoria, FluA/H1N1, FluA/H3N2, HAdV-B, HAdV-C, HAdV-E, HCoV-NL63, HCoV-229E, HCoV-OC43, HCoV-HKU1, SARS-CoV-2, VZV, CMV, and EBV (Supplementary Table S2b). To prepare each pool, equal volumes of 100 µM primer stock solutions were combined and subsequently diluted so that the concentration of each primer in the working pool was 2 µM. For each 25 µL multiplex PCR reaction, 0.625 µL of the corresponding primer pool was added, resulting in a final concentration of 0.05 µM for each primer.

Short conserved regions of the viral genomes were amplified in a total reaction volume of 25 µL containing 12.5 µL of Q5^®^ High-Fidelity 2X Master Mix (New England Biolabs, USA), 0.625 µL of primer mixture (Pool 1 or Pool 2), 2.5 µL of 20 mM MgCl_2_ (AmpliSens^®^, Russia), 2.375 µL of Milli-Q water (AmpliSens^®^, Russia), and 7 µL of DNA template. The addition of MgCl_2_ increased its concentration by 2 mM, resulting in a total final MgCl_2_ concentration of 4 mM when the MgCl_2_ already present in the Q5^®^ High-Fidelity 2X Master Mix was taken into account.

Each PCR run included one negative control for every 23 clinical specimens. The thermal cycling conditions were as follows: 98 °C for 30 s; and then 35 cycles of 98 °C for 10 s, 58 °C for Pool 1 or 60 °C for Pool 2 for 30 s, 72 °C for 40 s; followed by a final extension at 72 °C for 2 min.

### 2.6. Sequencing Library Preparation

After amplification, the reaction mixtures generated using the two primer pools were combined in equal volumes. The PCR products were purified using AMPure XP magnetic beads (Beckman Coulter, Brea, CA, USA) at a sample-to-bead volume ratio of 1:0.8. DNA was eluted in 15 µL of 0.1× TE buffer (AmpliSens^®^, Russia). Each primer in the panel contained a Nextera adapter sequence (Illumina) at its 5′ end; consequently, the resulting amplicons were flanked by adapter sequences, allowing direct progression to sequencing library construction.

Paired-end sequencing libraries were prepared by indexing PCR in a total reaction volume of 25 µL containing 10 µL of PCR-mix-2 blue (AmpliSens^®^, Russia), 2 µL of 4.4 mM dNTPs (T) (AmpliSens^®^, Russia), 1 µL of EvaGreen^®^ Dye supplied as a 20× aqueous solution (Biotium, Fremont, CA, USA), 2 µL of i7 index primer (5 pM/µL), 2 µL of i5 index primer (Illumina, USA; 5 µM), and 8 µL of purified amplicons. The barcoding reaction was performed under the following thermal cycling conditions: 98 °C for 30 s, followed by 10 cycles of 98 °C for 10 s and 65 °C for 1 min 15 s, with fluorescence acquisition at 65 °C.

The resulting libraries were purified using magnetic beads as described above. The concentrations of the purified libraries were measured using the Qubit dsDNA HS Assay Kit (Thermo Fisher Scientific, Waltham, MA, USA). The libraries were pooled in equimolar amounts. Double-sided size selection was performed in two steps using AMPure XP magnetic beads (Beckman Coulter, Brea, CA, USA) to remove off-target products. The fragment-size distributions of the final libraries were assessed using a 2100 Bioanalyzer automated electrophoresis system (Agilent Technologies, Santa Clara, CA, USA). The concentration of the final library pool was determined using the NEBNext Library Quant Kit for Illumina (New England Biolabs, Ipswich, MA, USA).

Sequencing was performed predominantly on an Illumina MiSeq platform using a MiSeq Reagent Kit v2, 300 cycles (Illumina, San Diego, CA, USA). Approximately 0.25% of the total sequencing reads were allocated to each specimen, corresponding to approximately 60,000 reads per specimen. A small subset of samples (*n* = 139 from Group I) was sequenced on the Salus Pro platform using PE100 (80 M) reagent kit (Salus BioMed, Shenzhen, China). Approximately 0.7% of total sequencing reads were allocated to each specimen, corresponding to approximately 600,000 reads per specimen.

### 2.7. Bioinformatics Data Analysis

To increase the reliability of the results and minimize algorithm-specific systematic errors, the sequencing data were analyzed in parallel using two independently developed bioinformatics pipelines (Pipelines I and II, described below). Pipeline I was based on mapping sequencing reads to reference consensus sequences of the target amplicons. Therefore, it enabled stringent verification that the reads aligned within the expected coordinates of the panel targets. Pipeline II was based on taxonomic classification of the reads using BLASTn against a custom precompiled database. This method provided broader coverage of viral genetic diversity by accounting for variation among reference sequences.

In most cases, a specimen was considered positive for a specific pathogen when concordant positive results were obtained with both pipelines. In cases of discordance, defined as a positive result obtained with only one pipeline, additional verification was performed. This included visual inspection of read alignments in the Integrative Genomics Viewer (IGV), analysis of the coverage distribution across the target amplicon, assessment of read quality, and evaluation of possible cross-contamination. The final classification was determined on the basis of all these criteria. Sample-level preliminary PCR results, outputs and read counts from both bioinformatics pipelines, and the final mp-tNGS interpretation are provided in Supplementary Table S5.

#### 2.7.1. Reference Sequence-Based Read-Mapping Analysis (Pipeline I)

Raw sequencing data were preprocessed using Trimmomatic to improve the accuracy of the subsequent analysis [27]. This process included adapter sequence removal, trimming of low-quality regions from the 5′ and 3′ ends of reads, and filtering of short reads. The processed reads were then mapped using Minimap2 to a reference database representing the viral pathogens targeted by the panel [28]. The database comprised consensus sequences corresponding to the target genomic regions of each viral pathogen included in the multiplex panel. Consensus sequences were generated by aligning viral genome sequences retrieved from NCBI using MAFFT [29,30]. For each pathogen, complete genomes from specimens collected in 2019 or later were selected. Because of the large number of available SARS-CoV-2 genomes, the most prevalent lineages circulating between 2022 and 2024 were selected, and three to five of the highest-quality sequences were included for each lineage. Multiple sequence alignment was then used to generate a representative consensus sequence for each pathogen. The specificity of the read-mapping analysis was increased by identifying the primer coordinates within each consensus sequence. Reads aligned within the interval bounded by the corresponding primer coordinates were therefore considered target-specific. SAMtools coverage was used to calculate the percentage and depth of coverage of the target genomic region and to count mapped reads located within the coordinates of the expected amplicons [31].

#### 2.7.2. Taxonomic Classification of Reads (Pipeline II)

Taxonomic classification of sequencing reads was performed using BLASTn [32]. The reference database used for the searches was constructed as follows. For all viral pathogens included in the primer panel, except SARS-CoV-2, the corresponding genomic sequences were retrieved from the NCBI core_nt database using the following taxonomic identifiers (taxids): 12063, 195055, 208893, 208895, 11226, 11224, 2560525, 12730, 11216, 11137, 277944, 31631, 290028, 108098, 129951, 130308, 689403, 573977, 638313, 114727, 119210, 11520, 10335, 10376, 10359, 3037664, and 3037665. Because of the large number of SARS-CoV-2 sequences in NCBI core_nt, 239 sequences from specimens collected in 2024–2025 were instead selected from the GISAID and VGARus databases [33,34]. The resulting dataset was manually filtered to retain only high-quality genomic sequences with high genome coverage. For each viral taxon, the region corresponding to the target amplicon was identified, after which the sequences were clustered using a 98% sequence identity threshold and manually curated. This procedure produced a representative sequence set encompassing the expected genetic diversity of the viral pathogens.

To enable the detection of potential contamination, genomic sequences corresponding to the following taxa were also added to the dataset: *Homo sapiens*, *Escherichia coli*, Moloney murine leukemia virus, Escherichia phage phiX174, and *Prevotella melaninogenica* ATCC 25845. The resulting sequence set was used to construct a custom BLAST v2.11.0 database with makeblastdb for the analysis of sequencing data generated using the multiplex panel [32].

During data analysis, the raw sequencing reads underwent several processing steps. First, quality control and adapter trimming were performed using Trimmomatic and Cutadapt [27,35]. Human-derived reads were then removed by mapping the reads to the human reference genome (GRCh38 assembly) using Bowtie 2 and retaining only the unmapped reads [36]. Next, overlapping paired-end reads were merged using the fastq_mergepairs function in VSEARCH to increase the sensitivity of subsequent classification, and the resulting reads were clustered using CD-HIT-EST at a 96% sequence identity threshold to accelerate database searches [37,38]. Finally, the clustered reads were queried against the custom database in megablast mode using an *E*-value threshold of 1 × 10^−6^.

After completion of the BLASTn search, a custom Python v.3.10.12 script was used to assign the corresponding taxon to each read. A preliminary read-count table was then generated, with taxa represented as rows, specimens as columns, and the number of reads assigned to each taxon reported in the corresponding cells. Initial filtering was subsequently applied: for each specimen, taxa supported by fewer than five reads were excluded.

When multiple specimens are sequenced simultaneously, contamination is a common problem, arising both from cross-contamination among specimens within the same sequencing run and from external sources. Consequently, a virus that is genuinely present and represented by a large number of reads in several specimens may also be detected at a low read count in other specimens in which the pathogen is actually absent. Because read counts can differ by several orders of magnitude among pathogens, a single fixed threshold is not applicable, and an adaptive threshold is required. We therefore applied an adaptive bioinformatics filtering procedure.

After initial filtering, a separate threshold was calculated for each sequencing run and each viral taxon. The threshold was defined as 1% of the total number of reads assigned to that taxon across all specimens within the run. Values below 50% of the threshold were excluded, whereas values ranging from 50% to 100% of the threshold were classified as potential contamination and subjected to additional manual review. Only values exceeding the threshold were retained for further interpretation. The value in each cell of the table was subsequently interpreted according to the following rules:

• If the value was less than half of the threshold for the corresponding taxon, it was replaced with 0;
• If the value ranged from half of the threshold to the full threshold for the corresponding taxon, it was retained but flagged as potential contamination;
• If the value exceeded the threshold for the corresponding taxon, it was retained unchanged.

This filtering strategy enables taxon-specific thresholds to be calculated adaptively and allows potential contamination to be either removed or flagged. The script also includes an optional additional filtering function that excludes taxa presumed to represent contaminants if they occur in more than a predefined, user-configurable percentage of the analyzed specimens or are present at very low abundance.

### 2.8. Assessment of Agreement Between Prior PCR and mp-tNGS Results

Agreement between PCR and mp-tNGS results was assessed using the specimens in Group I. Importantly, unlike our method, the PCR assays used for the initial diagnostic testing did not enable viral typing in all cases. For example, PCR did not differentiate adenoviruses into types B, C, and E, whereas the developed panel was capable of doing so. Therefore, to ensure a valid assessment of agreement between the two methods, comparisons were performed, where necessary, at higher taxonomic levels. HCoV-229E, HCoV-NL63 were aggregated as alpha-HCoV; HCoV-OC43 and HCoV-HKU1 were aggregated as beta-HCoV; HRSV-A and HRSV-B as HRSV; adenoviruses B, C, and E as HAdV; all parainfluenza viruses as HPIV; all bocaviruses as HBoV; HMPV-A and HMPV-B as HMPV; Influenza A and Influenza B were analyzed separately. For samples classified as Influenza A and Influenza B by tNGS, 6 samples were excluded from the comparison because the corresponding PCR result was reported only as “Influenza”, without subtype/species specification. Similarly, for samples classified as beta-HCoV and alpha-HCoV by tNGS, 4 samples were excluded because the corresponding PCR result was reported only as “HCoV”. EBV, VZV, and CMV were excluded from the comparative analysis because they were not among the targets of the prior PCR assays. Similarly, SARS-CoV-2 was excluded because it was represented only among the mp-tNGS targets. For each comparison category, a 2 × 2 contingency table was constructed, and overall percent agreement (OPA), positive percent agreement (PPA) and Cohen’s kappa coefficient were calculated. For the calculation of PPA, prior PCR results were used as the reference, and mp-tNGS results were evaluated against them. PPA was calculated as the proportion of PCR-positive specimens that were also positive by mp-tNGS [TP/(TP + FN) × 100%].

### 2.9. Assessment of the Analytical Sensitivity of the Multiplex Panel

The analytical sensitivity of the multiplex primer panel was evaluated using specially developed positive control samples (PCSs) representing encapsulated RNA or DNA. The DNA-containing PCSs consisted of a recombinant λ phage based on the λgt10 vector, into which a short viral genomic region—the target amplicon had been cloned. Recombinant MS2 bacteriophages based on the pET20-ms2 Xcm-stuff vector and carrying the corresponding target amplicons as inserts were used as the RNA-containing PCSs. The primers used to generate these amplicons did not contain a Nextera adapter at their 5′ ends. In total, PCSs were generated for 23 of the 28 viral targets. Control samples could not be generated for HPIV-4b, HBoV-2, HBoV-3, HPeV-1, or HPeV-3 because no biological material containing these viruses was available in the laboratory.

The initial concentrations of the PCSs were determined by quantitative PCR using assays targeting gene regions of the corresponding bacteriophages. To optimize the experimental workload, the analytical sensitivity, expressed as the limit of detection (LoD), was evaluated using six control panels (Supplementary Table S3) [26]. Each panel consisted of a mixture of several PCSs, with each recombinant phage present at the same initial concentration of 1 × 10^6^ copies/mL (Supplementary Table S3). Each control panel was then serially diluted to obtain concentrations of 1 × 10^5^, 5 × 10^4^, 1 × 10^4^, 5 × 10^3^, and 1 × 10^3^ copies/mL. To simulate clinical specimens, the control panels were serially diluted in a negative clinical matrix consisting of pooled nasopharyngeal and oropharyngeal swab specimens in transport medium. The negative matrix had previously been confirmed to be free of the target viruses using the registered PCR assays ARVI-screen-short, EBV/CMV/HHV6A/B-screen-FL, and VZV-FL (AmpliSens^®^). The dilution series and negative controls consisting of the negative clinical matrix were tested in four replicates, processed through the entire sample-preparation workflow, and sequenced as described above.

## 3. Results

### 3.1. Optimization of Amplification Conditions

The performance of the primer panel was initially evaluated using a small number of Group I specimens containing previously identified viruses. We initially assessed whether the primers could perform as a single multiplex pool. Preliminary experiments performed using a small set of positive specimens demonstrated extensive primer-dimer formation and inadequate amplification of the intended targets. The panel was therefore divided into two primer pools on the basis of predicted heterodimer interactions. Heterodimer formation was assessed using the predicted Gibbs free energy (ΔG). Primers involved in interactions with ΔG values of ≤ −10 kcal/mol were assigned to separate pools. Thus, predicted interactions between primers retained within the same pool had ΔG values above −10 kcal/mol. This separation enabled successful amplification of most target regions during retest of the preliminary specimen set. However, we observed persistent amplification problems for HRSV-A, HRSV-B, FluB/Victoria, HCoV-NL63, and SARS-CoV-2. For these targets, alternative candidate primer pairs were designed and evaluated in singleplex and multiplex reactions. The best-performing primer pairs were subsequently incorporated into the panel. Multiplex PCR conditions were optimized using annealing-temperature gradients ranging from 55 °C to 65 °C for each pool and final concentrations of each primer in reaction ranging from 0.025 to 0.075 µM. The highest overall yield of target amplicons was obtained at annealing temperatures of 58 °C for Pool 1 and 60 °C for Pool 2, with a final concentration of 0.05 µM of each primer for both pools. To increase template concentration, the nucleic-acid elution volume was reduced from the volume recommended by the extraction-kit manufacturer to 25 µL. The volume of template added to each multiplex PCR reaction was also increased. Since HMPV amplification remained insufficient, the MgCl_2_ concentration was additionally optimized. After considering established approaches to multiplex PCR optimization, we focused on determining the optimal concentration of Mg^2+^ ions in the reaction mixture [39]. According to the manufacturer’s instructions, the initial MgCl_2_ concentration in the PCR master mix used in this study was 2 mM. The MgCl_2_ concentration was experimentally increased from the initial value to 3, 3.5, and 4 mM. A marked improvement in the amplification of the target HMPV genomic regions was observed at MgCl_2_ concentrations of 3.5 and 4 mM. On the basis of these findings, a final MgCl_2_ concentration of 4 mM was selected for enrichment of the target viral genomic fragments using the multiplex panel.

### 3.2. Analytical Sensitivity Results

Before testing a large number of specimens, the limit of detection (LoD) of the developed panel was evaluated for 23 of 28 target pathogens. Five targets, comprising HPIV-4b, HBoV-2, HBoV-3, HPeV-1 and HPeV-3 were excluded from this experiment due to the absence of biological samples containing them at our disposal. Analytical sensitivity was assessed by amplification and sequencing of control panels containing encapsulated positive control samples (PCSs) over a concentration range of 1 × 10^5^ to 1 × 10^3^ copies/mL. Each concentration was tested in four independent replicates. The dilution series included negative controls consisting of the negative clinical matrix to detect potential cross-contamination between high- and low-copy-number dilutions. The negative controls were sequenced together with the PCSs. Probit regression was used to estimate the limit of detection. The model input comprised the number of successfully detected PCS replicates at each concentration (Supplementary Table S4), with the negative controls treated as the zero-concentration level (Supplementary Table S4). The resulting LoD estimates therefore represent the pathogen concentration at which the mp-tNGS method is expected to yield a positive result with a probability of 95% [26].

The estimated limit of detection was 6.3 × 10^2^ copies/mL for 10 of the 23 targets evaluated. For nine targets, the estimated LoD ranged from 1.1 × 10^3^ to 9.4 × 10^3^ copies/mL, depending on the virus. For HPIV-1, HCoV-HKU1, HMPV-B, and HMPV-A, the estimated LoDs were 7.5 × 10^4^, 7.3 × 10^4^, 7.0 × 10^4^, and 1.1 × 10^4^ copies/mL, respectively. Detailed LoD estimates are presented in Table 2. Because the LoD experiments were performed using control panels containing mixtures of positive control samples rather than testing each PCS separately, the actual analytical sensitivity of the panel for individual viral targets may differ slightly from these estimates.

### 3.3. Detection of Viral Targets in Clinical Specimens

The study groups were defined according to the range of pathogens detectable using our mp-tNGS method. Therefore, specimens containing human rhinoviruses or bacterial pathogens were excluded.

Group I was used to assess agreement between mp-tNGS and PCR results. This group comprised 216 specimens that had previously tested positive by PCR for at least one virus corresponding to a target included in the developed multiplex primer panel. PCR and mp-tNGS results were fully concordant in 139 specimens (64%). In 28 specimens (13%), mp-tNGS detected one or more additional viruses beyond those identified by PCR. Most of these additional findings represented coinfections involving herpesviruses. In two specimens (<1%) with coinfections previously identified by PCR, mp-tNGS detected only one of the pathogens. In one further specimen (<1%), mp-tNGS likewise detected only one of the pathogens previously identified by PCR and, in addition, detected a virus that had not been covered by the prior testing. In the remaining 46 specimens (21%), mp-tNGS did not detect the virus identified during prior PCR testing. The reasons for these discordant results could not be definitively established because Ct values from the initial PCR testing were unavailable. However, the relatively high LoDs observed for several targets indicate that insufficient analytical sensitivity for individual targets may have contributed substantially to the discordant results. Other possible factors include low target abundance, nucleic acid degradation during transportation and storage, primer–target mismatches, and the applied bioinformatics thresholds. Nevertheless, other viral targets were identified in six of these 46 specimens.

**Figure 1:**
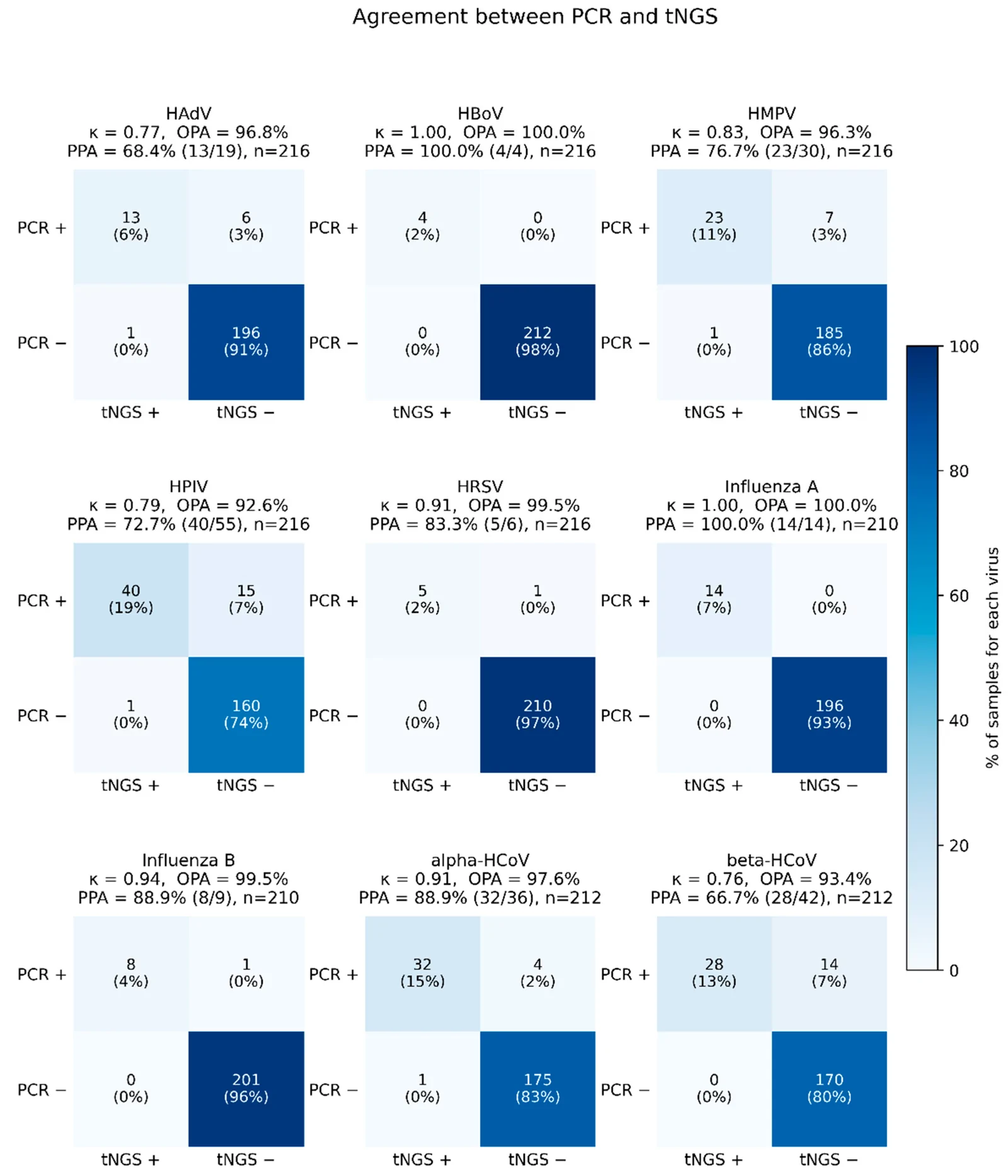
Agreement between mp-tNGS and PCR results based on the analysis of Group I specimens. For each taxon, *n* indicates the number of specimens included in the corresponding comparison. OPA, overall percent agreement; PPA, positive percent agreement; *κ*, Cohen’s kappa coefficient, a measure of agreement between two methods that accounts for agreement expected by chance. Values of κ closer to 1 indicate stronger agreement beyond that expected by chance.

Since the prior PCR did not define types or subtypes for several viruses, concordance between the two methods could be evaluated only at higher taxonomic levels. Therefore, it was impossible to evaluate target-specific concordance for individual mp-tNGS primer pairs. To assess agreement between PCR and mp-tNGS, nine 2 × 2 contingency tables were constructed, one for each of the nine aggregated taxa (Figure 1). The overall percent agreement (OPA) was at least 92.6% for all taxa, while positive percent agreement (PPA) varied from 66.7% to 100%. Cohen’s kappa coefficient exceeded 0.76 for every taxon, indicating substantial agreement between the methods.

To demonstrate the utility of mp-tNGS, compared with conventional PCR, for screening large numbers of specimens, a second study group (Group II) was established. This group comprised specimens that had tested negative during prior PCR-based diagnostic testing. Importantly, the initial screening preceding this study was performed using assays with a comparatively narrower range of targets. Therefore, a negative result in the prior PCR testing did not indicate the absence of all viruses targeted by our panel. Accordingly, viruses detected by mp-tNGS should not be regarded as direct discrepancies with the PCR results but rather as findings resulting from the broader range of viral targets analyzed. Group II comprised 394 presumptively negative specimens, of which 81 tested positive by mp-tNGS. Coinfections involving EBV were identified in three of these specimens.

**Figure 2:**
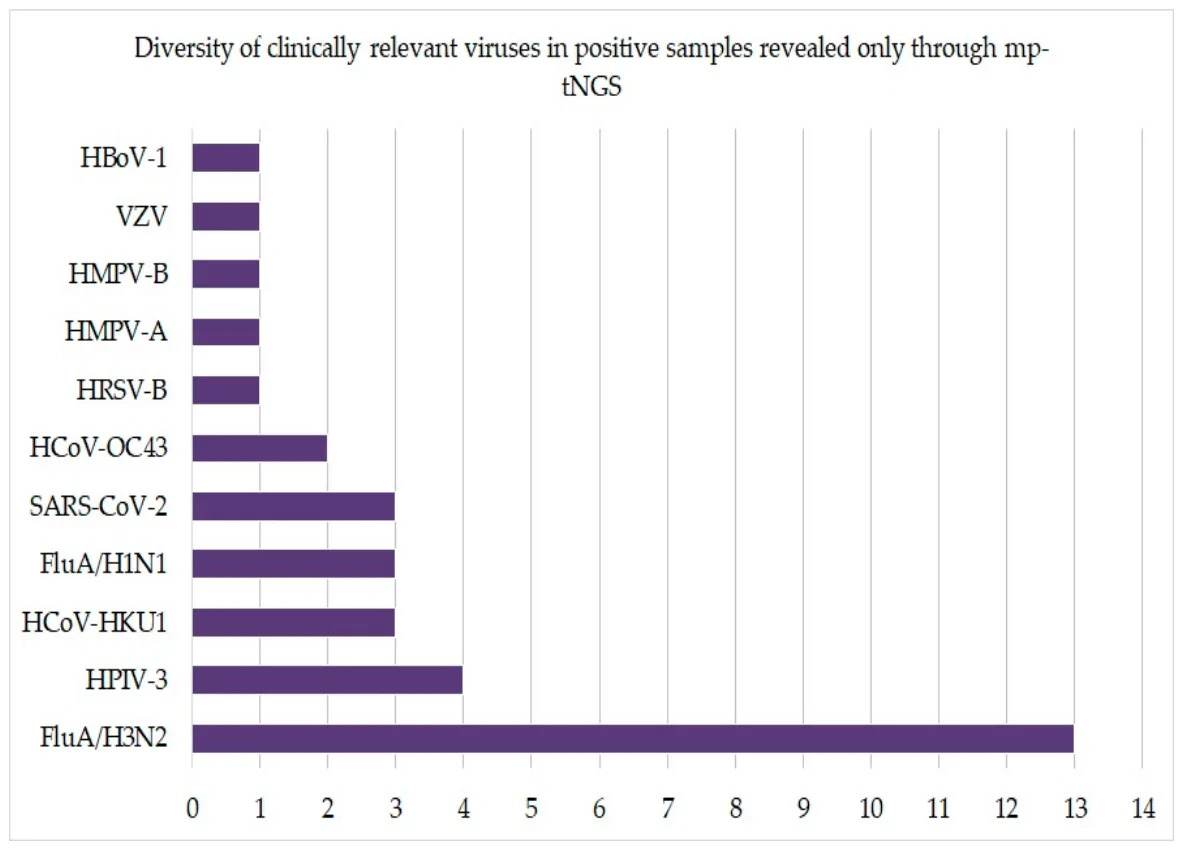
Distribution of clinically relevant viruses detected by mp-tNGS among Group II specimens. EBV detections (*n* = 51) are not shown and are discussed separately.

Epstein–Barr virus (EBV) was the most frequently detected viral target in Group II (*n* = 51). Because the assay was non-quantitative and no additional clinical, serological, or blood-based molecular data were available, these findings could not distinguish latent shedding from reactivation or active infection. Accordingly, EBV detections were analyzed descriptively and were not regarded as evidence of an etiological role in the current respiratory episode. At the same time, mp-tNGS identified several other pathogens of greater clinical relevance in Group II, including FluA/H3N2 (*n* = 13), HPIV-3 (*n* = 4), HCoV-HKU1 (*n* = 3), FluA/H1N1 (*n* = 3), SARS-CoV-2 (*n* = 3), and HCoV-OC43 (*n* = 2) (Figure 2). In addition, single cases of infection with HRSV-B, HMPV-A, HMPV-B, VZV, and HBoV-1 were detected. Collectively, these findings illustrate the additional detection yield associated with the broader target coverage of mp-tNGS in specimens with negative results from prior, more narrowly targeted PCR testing. These findings should not be interpreted as evidence of superior analytical sensitivity compared with conventional PCR. Instead, they highlight that expansion of the detectable pathogens range enables the identification of viruses that might have been missed by conventional molecular diagnostic methods (Figure 2).

**Figure 3:**
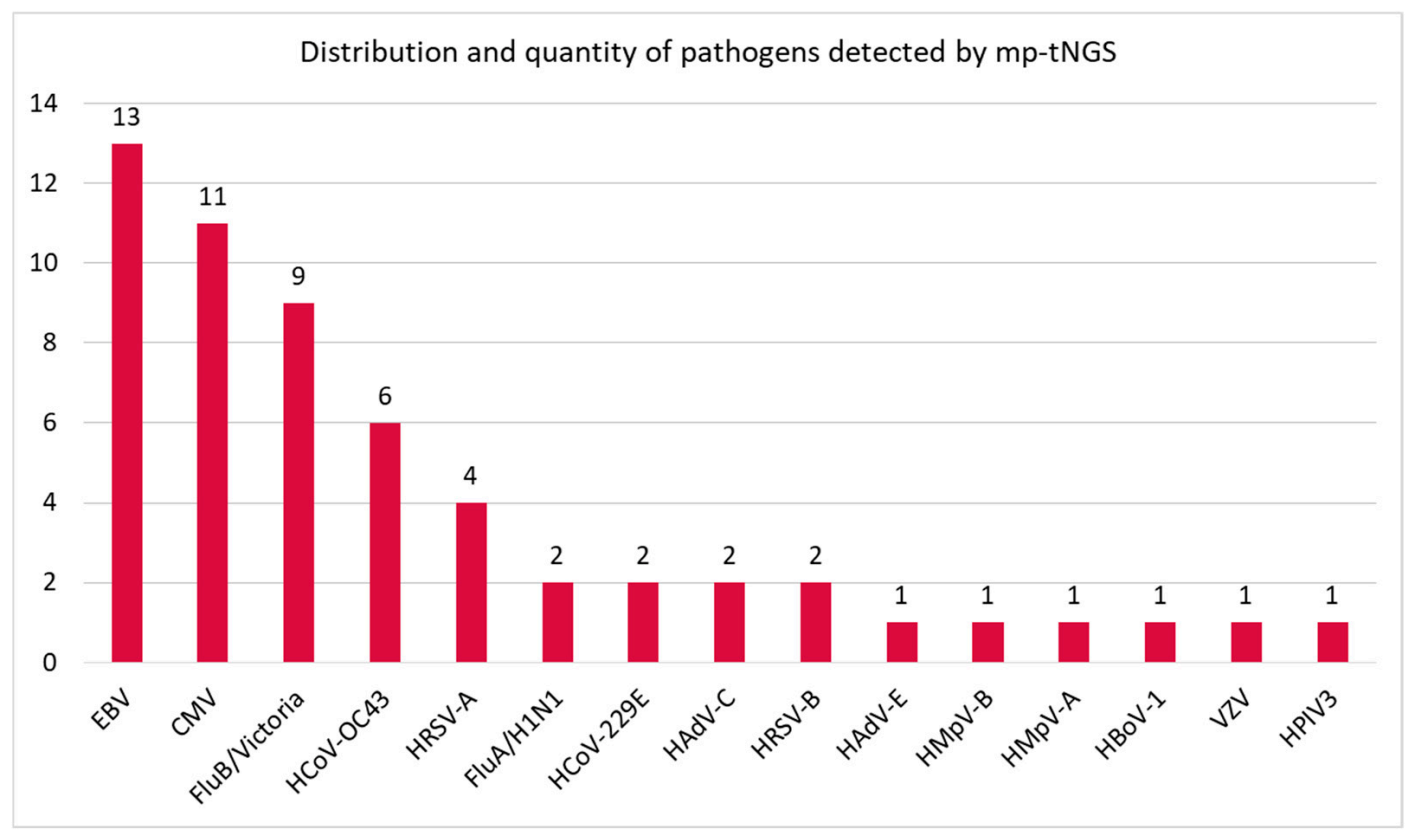
Distribution and number of viruses uniquely detected by mp-tNGS in Group III.

Group III comprised 300 specimens obtained from hospitalized patients with symptoms of acute respiratory infection. According to the results of prior PCR testing, a viral pathogen had been identified in 95 of these specimens. In Group III, mp-tNGS detected at least one viral target in 131 specimens. Complete concordance between the mp-tNGS and PCR results was observed in 64 specimens. In seven PCR-positive specimens, mp-tNGS additionally detected viruses belonging to the family *Herpesviridae*, namely EBV or CMV. In five specimens, mp-tNGS detected a pathogen different from that identified during the prior PCR testing. In one specimen, mp-tNGS detected only one of the two viruses previously identified by PCR. In 18 specimens, however, the PCR findings were not confirmed by mp-tNGS. At the same time, mp-tNGS identified viral infections not detected by the prior PCR testing in 54 specimens (Figure 3).

Overall, the developed panel enabled the detection of viruses that had been missed during the prior testing and identified nine additional cases of coinfection beyond those detected by PCR. Consequently, the total number of specimens with confirmed coinfections increased to 11. However, a substantial proportion of the additional mp-tNGS findings involved EBV and CMV and therefore require cautious clinical interpretation. At the same time, the sensitivity of the panel appeared to be insufficient to confirm the PCR findings in several specimens.

## 4. Discussion

In this study, we developed and evaluated a multiplex primer panel for the detection of viral pathogens and selected viral subtypes, most of which are associated with respiratory tract infections. Our findings suggest that this approach could serve as an adjunct to routine PCR-based diagnostics, particularly given the limited number of targets that can be detected simultaneously using conventional PCR assays. The potential contribution of mp-tNGS to patient management lies in its ability to identify a broader range of candidate pathogens in cases with negative or inconclusive initial testing, detect co-infections, and provide viral type or subtype information. When interpreted together with clinical findings, these results may support subsequent diagnostic, infection-control, and, for clinically actionable pathogens, treatment decisions. However, the present study did not evaluate whether mp-tNGS findings resulted in changes in patient management or clinical outcomes, and this will require prospective clinical assessment.

The developed panel detected both viruses previously identified by PCR and additional viral targets, including coinfections. More broadly, tNGS-based approaches for the detection and characterization of respiratory viruses represent a promising complement to routine diagnostics by expanding the range of detectable pathogens and providing additional genetic information.

Some pathogens identified during prior PCR testing were not detected using our mp-tNGS method. Despite the multistep optimization of the multiplex assay, some variability in analytical sensitivity remained. The relatively high LoDs observed for HPIV-1, HCoV-HKU1, HMPV-A, and HMPV-B may have contributed to the non-confirmation of some prior PCR findings, although this could not be assessed directly because the initial Ct values were unavailable for most specimens. Other possible but unconfirmed factors include low target concentrations, storage-related nucleic acid degradation, primer–target mismatches, and the applied bioinformatics thresholds. Conventional PCR assays targeting one or a few targets generally have greater sensitivity at low viral loads because they contain relatively few primers in the reaction mixture and generate short amplicons. By contrast, mp-tNGS results depend not only on the quantity and integrity of the starting material but also on the amplification efficiency of numerous primer pairs within a single reaction, sequencing depth, and the distribution of reads among individual targets. Although bioinformatics read filtering reduces the risk of false-positive results associated with cross-contamination and index hopping, it may also reduce sensitivity in specimens with low target concentrations. It is worth mentioning that we did not perform an individual primer concentration balancing during this study. We assume that such procedures, alongside the redesign of some selected primers, could improve the uniformity of analytical sensitivity.

Additional measures for contamination control in mp-tNGS may include the use of unique molecular identifiers (UMIs) introduced during amplification or library preparation [40], as well as filtering thresholds that account for reads detected in negative controls.

The interpretation of herpesvirus findings requires particular attention. EBV and CMV were frequently detected in respiratory specimens; however, the presence of their DNA in upper respiratory tract specimens does not necessarily indicate active infection or establish an etiological role in the current episode of acute respiratory infection. Herpesviruses can persist in the body for prolonged periods and reactivate under various conditions, particularly in immunocompromised patients. Therefore, the detection of EBV, CMV, or VZV by mp-tNGS should be regarded as a potentially clinically relevant finding, but its interpretation should take into account the patient’s symptoms and immune status.

The panel presented in this study was designed to cover 28 viral targets, of which 23 underwent experimental analytical evaluation. The remaining five targets were assessed in silico only and are not included in the experimentally supported analytical performance claims. Most of the represented pathogens associated with respiratory infections. Additionally, our panel covers VZV, CMV, and EBV, which may be clinically relevant in specific patient groups. Nevertheless, several clinically important respiratory pathogens were not included in the panel. These primarily include human rhinoviruses (HRVs) and human enteroviruses (EVs), which exhibit substantial genetic diversity, complicating the design of universal primers that also provide sufficient sequence information for viral typing. HSV-1, HSV-2, and HHV-6 were not included in the current version of the panel because their detection was not among the primary objectives of this study. Moreover, their detection in respiratory specimens may be difficult to interpret due to the high prevalence of latent infection and viral reactivation.

Despite the complexity of primer design for a multiplex panel and the need for careful selection of read-filtering thresholds, mp-tNGS offers several potential advantages. The method enables the simultaneous detection of a broad range of viral targets without requiring numerous separate PCR reactions. This approach may be useful in cases of infection with an undetermined etiology, given that the suspected causative agent is included among the panel targets; when coinfection is suspected; in patients with severe or atypical disease; and during investigations of localized outbreaks. Furthermore, unlike many PCR panels, for the experimentally evaluated targets, mp-tNGS can also provide viral type or subtype information that may be useful for epidemiological analysis.

Nevertheless, widespread implementation of mp-tNGS will require interlaboratory standardization of specimen validity criteria, minimum read-count thresholds, coverage depth and uniformity, acceptable levels of background reads, and rules for interpreting equivocal positive results supported by low pathogen read counts. Standardization of bioinformatics processing is particularly important because the selection of filtering thresholds directly affects the balance between sensitivity and specificity.

Although a formal economic evaluation was beyond the scope of this study, the cost of NGS has fallen substantially in recent years because of increased sequencing throughput, improved library preparation methods, and the ability to multiplex larger numbers of specimens in a single run. The per-specimen cost of mp-tNGS depends strongly on the sequencing platform, batch size, degree of automation, and number of pathogens that would otherwise require separate PCR assays. When sequencing runs are fully loaded and broad pathogen coverage is required, the cost difference between mp-tNGS and multiple separate PCR tests may be reduced. However, implementation also involves costs associated with library preparation and quality control, computational infrastructure, data storage, validated bioinformatics pipelines, and trained laboratory personnel. Before routine diagnostic use, the method will require further analytical and clinical validation, standardized quality-control and interpretation criteria, and prospective assessment of its cost-effectiveness and clinical impact.

Several limitations of this study should be acknowledged. The principal limitations included incomplete equivalence between the target ranges of the prior PCR panels and the developed mp-tNGS panel, the absence of positive control samples for five viruses as well as clinical samples containing them, and limited clinical information. These factors hindered direct assessment of the diagnostic sensitivity of the method and the clinical significance of some of the additional findings.

Further development of the panel may include the addition of HRVs, EVs, and bacterial respiratory pathogens, such as *Mycoplasma pneumoniae*, *Chlamydia pneumoniae*, *Bordetella pertussis*, and other pathogens relevant to differential diagnosis. In addition, expanding the number of targeted genomic regions may provide more comprehensive information on genotypes, lineages, subtypes, and individual mutations of epidemiological or clinical relevance.

Thus, the developed mp-tNGS approach may be regarded as an intermediate approach between routine multiplex PCR and metagenomic sequencing. Compared with PCR, it provides broader coverage of known pathogens and additional information on the viruses detected. Compared with metagenomic sequencing, mp-tNGS preferentially enriches preselected targets and may therefore reduce the required sequencing depth and cost of analysis; however, it is not intended for the detection of unknown pathogens. This balance of advantages and limitations is generally consistent with the current understanding of the clinical applications of mNGS- and tNGS-based approaches in infectious disease diagnostics, in which the choice of method is determined by the purpose of the investigation, the expected range of pathogens, sensitivity requirements, and available resources [21]. The present findings support further development of mp-tNGS as a potential adjunctive tool for expanded laboratory testing and selected molecular epidemiological applications. Further optimization and prospective clinical validation are required before routine diagnostic implementation.

## Figures and Tables

**Table 1:** Viral targets included in the multiplex primer-panel design.

№	Viral Target/Taxon	Panel Designation
1	Severe acute respiratory syndrome coronavirus 2	SARS-CoV-2
2	Influenza A virus, subtype H1N1	FluA/H1N1
3	Influenza A virus, subtype H3N2	FluA/H3N2
4	Influenza B virus, Victoria lineage	FluB/Victoria
5	Human orthopneumovirus, subgroup A/Respiratory syncytial virus A	HRSV-A
6	Human orthopneumovirus, subgroup B/Respiratory syncytial virus B	HRSV-B
7	Human metapneumovirus, subgroup A	HMPV-A
8	Human metapneumovirus, subgroup B	HMPV-B
9	Human coronavirus NL63	HCoV-NL63
10	Human coronavirus 229E	HCoV-229E
11	Human coronavirus OC43	HCoV-OC43
12	Human coronavirus HKU1	HCoV-HKU1
13	Human respirovirus 1/Human parainfluenza virus 1	HPIV-1
14	Human orthorubulavirus 2/Human parainfluenza virus 2	HPIV-2
15	Human respirovirus 3/Human parainfluenza virus 3	HPIV-3
16	Human orthorubulavirus 4a/Human parainfluenza virus 4a	HPIV-4a
17	Human orthorubulavirus 4b/Human parainfluenza virus 4b	HPIV-4b
18	Human mastadenovirus B	HAdV-B
19	Human mastadenovirus C	HAdV-C
20	Human mastadenovirus E	HAdV-E
21	Human bocavirus 1	HBoV-1
22	Human bocavirus 2	HBoV-2
23	Human bocavirus 3	HBoV-3
24	Human parechovirus 1	HPeV-1
25	Human parechovirus 3	HPeV-3
26	Varicella-zoster virus	VZV
27	Human betaherpesvirus 5/Cytomegalovirus	CMV
28	Human gammaherpesvirus 4/Epstein–Barr virus	EBV

**Table 2:** LoD estimates for 23 viral targets calculated using probit regression.

Pathogen	LoD (Copies/mL)
HPIV-1	75,444
HCoV-HKU1	72,727
HMPV-B	69,598
HMPV-A	10,515
HRSV-B	9423
HCoV-OC43	7153
HRSV-A	7130
HCoV-NL63	6067
FluB/Victoria	5570
FluA/H3N2	3528
HCoV-229E	1271
HPIV-2	1144
HPIV-4a	1144
HBoV-1	633
HAdV-B	633
HAdV-C	633
HAdV-E	633
VZV	633
CMV	633
EBV	633
HPIV-3	633
FluA/H1N1	633
SARS-CoV-2	633

## Data Availability

The data presented in this study are available on request from the corresponding author because data was generated using human clinical specimens and may contain residual human-derived sequencing reads. Processed data supporting the findings of this study, including sample-level prior PCR results, outputs and read counts from bioinformatics pipelines, are provided in Supplementary Table S5.

## Supplementary Materials

The following supporting information can be downloaded at: https://www.mdpi.com/article/10.3390/diagnostics16182888/s1, Table S1: List of primers forming the multiplex panel; Table S2a: Composition of primer pool 1; Table S2b: Composition of primer pool 2; Table S3: Composition of the control panels; Table S4: Analytical sensitivity of mp-tNGS; Supplementary Table S5: ample-level comparison of preliminary PCR results and mp-tNGS findings generated by two independent bioinformatics pipelines.
