# Supplementary material for: Development and Initial Analytical Evaluation of a Multiplex PCR-Based Targeted NGS Assay for Expanded Viral Detection in Clinical Respiratory Specimens
Source: Diagnostics (Basel). 2026 Sep 8;16(18):2888. doi: 10.3390/diagnostics16182888 (PMC13605325; doi:10.3390/diagnostics16182888)
Supplement: Supplementary file 1 [file diagnostics-16-02888-s001.zip › Tables S1-S4.pdf]

Table S1. List of primers forming the multiplex panel

Primer name	Primer sequence with Nextera adapter (Nextera adapter + **specific primer sequence** 5' → 3')
Parechovirus_1_F	TCGTCGGCAGCGTCAGATGTGTATAAGAGACAG**AAGCCTCAATTGTAACAAAACA**
Parechovirus_1_R	GTCTCGTGGGCTCGGAGATGTGTATAAGAGACAG**TCACCAGACGTCTCACAAA**
Parechovirus_3_F	TCGTCGGCAGCGTCAGATGTGTATAAGAGACAG**CGTGCCATAACCCTAATAGT**
Parechovirus_3_R	GTCTCGTGGGCTCGGAGATGTGTATAAGAGACAG**GCAGGTACTAAATGTCTTCCAT**
Parainfluenza_1_F	TCGTCGGCAGCGTCAGATGTGTATAAGAGACAG**AACTAAGGTGTGAGACTACG**
Parainfluenza_1_R	GTCTCGTGGGCTCGGAGATGTGTATAAGAGACAG**CCAAATCAACATCTATCACAGT**
Parainfluenza_2_F	TCGTCGGCAGCGTCAGATGTGTATAAGAGACAG**TGTTTAACAATTCCCAAGTCA**
Parainfluenza_2_R	GTCTCGTGGGCTCGGAGATGTGTATAAGAGACAG**ATTAGCAGGGCAAAAACTTG**
Parainfluenza_3_F	TCGTCGGCAGCGTCAGATGTGTATAAGAGACAG**ACACATCAAAACAGGGGT**
Parainfluenza_3_R	GTCTCGTGGGCTCGGAGATGTGTATAAGAGACAG**GCTAGTGTTTTTGATATAGATCC**
Parainfluenza_4a_F	TCGTCGGCAGCGTCAGATGTGTATAAGAGACAG**CAGATAACATCAATCGCCTTA**
Parainfluenza_4a_R	GTCTCGTGGGCTCGGAGATGTGTATAAGAGACAG**TATTAGAGTTTGCAAGATCTCG**
Parainfluenza_4b_F	TCGTCGGCAGCGTCAGATGTGTATAAGAGACAG**TCTTTCTGAAGATTGTGAAATTT**
Parainfluenza_4b_R	GTCTCGTGGGCTCGGAGATGTGTATAAGAGACAG**AGCTTTATTTCCTGGGCAATC**
hMpv_A_F	TCGTCGGCAGCGTCAGATGTGTATAAGAGACAG**AAGTTAGCATGGACAGACAAA**
hMpv_A_R	GTCTCGTGGGCTCGGAGATGTGTATAAGAGACAG**TTGTCTGAAAGCAAGTCTAG**
hMpv_B_F	TCGTCGGCAGCGTCAGATGTGTATAAGAGACAG**TTCTTTACTTTATTGGAGAAGGA**
hMpv_B_R	GTCTCGTGGGCTCGGAGATGTGTATAAGAGACAG**AAGCATCTTTACTTATTCTGTGT**
hBoca1_F	TCGTCGGCAGCGTCAGATGTGTATAAGAGACAG**ACTTTCTTTAACACTTGGCA**
hBoca1_R	GTCTCGTGGGCTCGGAGATGTGTATAAGAGACAG**TGAGGTCTTCGAAGCAG**
hBoca2_F	TCGTCGGCAGCGTCAGATGTGTATAAGAGACAG**TCCTGTCAGGGAAGATATTT**
hBoca2_R	GTCTCGTGGGCTCGGAGATGTGTATAAGAGACAG**CATCACAAGGCCTAGGA**
hBoca3_F	TCGTCGGCAGCGTCAGATGTGTATAAGAGACAG**TGCAAAAACATCATGTCCTC**
hBoca3_R	GTCTCGTGGGCTCGGAGATGTGTATAAGAGACAG**GGTAGTTTTTGAAGAAGCGAA**
RSV_A_v6_F	TCGTCGGCAGCGTCAGATGTGTATAAGAGACAG**AAGAAATGGGAGAGGTGGCT**

Primer name	Primer sequence with Nextera adapter (Nextera adapter + **specific primer sequence** 5' → 3')
RSV_A_v6_R	GTCTCGTGGGCTCGGAGATGTGTATAAGAGACAG**GACCTGATCTATCTCCTGCTG**
RSV_B_1023_F	TCGTCGGCAGCGTCAGATGTGTATAAGAGACAG**CAATCATCCACAAGAGGGGG**
RSV_B_1023_R	GTCTCGTGGGCTCGGAGATGTGTATAAGAGACAG**CAGCTTCTCCTCCCAACTTC**
Bvic_v5_F	TCGTCGGCAGCGTCAGATGTGTATAAGAGACAG**AGTTACACTGTTGGTTTGGTGGG**
Bvic_v5_R	GTCTCGTGGGCTCGGAGATGTGTATAAGAGACAG**TTGTTGCTGTTGTTCCCATTCCT**
InfA-N1-F	TCGTCGGCAGCGTCAGATGTGTATAAGAGACAG**AGTGATGGACAGGCCTCATA**
InfA-N1-R	GTCTCGTGGGCTCGGAGATGTGTATAAGAGACAG**CCCCACTGCATATGTATCCC**
InfA-N2-F	TCGTCGGCAGCGTCAGATGTGTATAAGAGACAG**GTGTTCCTTTCCATCTGGGG**
InfA-N2-R	GTCTCGTGGGCTCGGAGATGTGTATAAGAGACAG**AAGTTCCATTGATACAAACGCA**
HAdV-B_F	TCGTCGGCAGCGTCAGATGTGTATAAGAGACAG**GGAGCAGAGATCTTGAAATC**
HAdV-B_R	GTCTCGTGGGCTCGGAGATGTGTATAAGAGACAG**TGATCCCCATTCATTGCGA**
HAdV-C_F	TCGTCGGCAGCGTCAGATGTGTATAAGAGACAG**AACCCCACGCACGAGAA**
HAdV-C_R	GTCTCGTGGGCTCGGAGATGTGTATAAGAGACAG**ACTCAGGAAGGCGTTTA**
HAdV-E_F	TCGTCGGCAGCGTCAGATGTGTATAAGAGACAG**AGGCGTGGTCCTGACAC**
HAdV-E_R	GTCTCGTGGGCTCGGAGATGTGTATAAGAGACAG**TGCTCCTACACCATCAACA**
NL63_v3_F	TCGTCGGCAGCGTCAGATGTGTATAAGAGACAG**CGTTGTGCTGTTACTGATGCC**
NL63_v3_R	GTCTCGTGGGCTCGGAGATGTGTATAAGAGACAG**CGAGTACGTGTGTCAAAACGACA**
229E_F	TCGTCGGCAGCGTCAGATGTGTATAAGAGACAG**GGTTCATGAGGTCATAATTGACA**
229E_R	GTCTCGTGGGCTCGGAGATGTGTATAAGAGACAG**TACAAATTACATGGTTCTAAGCACA**
OC43_F	TCGTCGGCAGCGTCAGATGTGTATAAGAGACAG**GGCTGTATGATGAATGTTGCT**
OC43_R	GTCTCGTGGGCTCGGAGATGTGTATAAGAGACAG**GAATAGTACCAGCAGGCAACC**
HKU1_F	TCGTCGGCAGCGTCAGATGTGTATAAGAGACAG**TTATGCTGAGGTTCATGCTG**
HKU1_R	GTCTCGTGGGCTCGGAGATGTGTATAAGAGACAG**GCAAGCCATACAGACTGCTT**
SARS-CoV-2_G1_F	TCGTCGGCAGCGTCAGATGTGTATAAGAGACAG**AGGTACGGCGCCGATCTAAA**
SARS-CoV-2_G1_R	GTCTCGTGGGCTCGGAGATGTGTATAAGAGACAG**TAGCGAGTGTATGCCCCTCC**

Primer name	Primer sequence with Nextera adapter (Nextera adapter + specific primer sequence 5' → 3')
VZV_F	TCGTCGGCAGCGTCAGATGTGTATAAGAGACAG**CTTTGATCTTCAAGGGCGAC**
VZV_R	GTCTCGTGGGCTCGGAGATGTGTATAAGAGACAG**CGCTATAGTTTGTCCCGGT**
CMV_ex2_F	TCGTCGGCAGCGTCAGATGTGTATAAGAGACAG**CGTAGGCCGCGTCAAAATA**
CMV_ex2_R	GTCTCGTGGGCTCGGAGATGTGTATAAGAGACAG**GCAGCTGGCCTTTTACAATA**
EBV_F	TCGTCGGCAGCGTCAGATGTGTATAAGAGACAG**TTTTAACATTGGCAGCAGGTAAG**
EBV_R	GTCTCGTGGGCTCGGAGATGTGTATAAGAGACAG**CAAATCAGGAGAACCACTGTAAAA**

Table S2a. Composition of primer pool 1

Primer name	Volume of 100 pM primer stock, µl	Primer concentration in pool, pM/µl	Final concentration of primers in the working pool, pM/µl
Parechovirus_1_F	2,5	3,57	2
Parechovirus_1_R	2,5	3,57	2
Parechovirus_3_F	2,5	3,57	2
Parechovirus_3_R	2,5	3,57	2
Parainfluenza_1_F	2,5	3,57	2
Parainfluenza_1_R	2,5	3,57	2
Parainfluenza_2_F	2,5	3,57	2
Parainfluenza_2_R	2,5	3,57	2
Parainfluenza_3_F	2,5	3,57	2
Parainfluenza_3_R	2,5	3,57	2
Parainfluenza_4a_F	2,5	3,57	2
Parainfluenza_4a_R	2,5	3,57	2
Parainfluenza_4b_F	2,5	3,57	2
Parainfluenza_4b_R	2,5	3,57	2
hMpv_A_F	2,5	3,57	2
hMpv_A_R	2,5	3,57	2
hMpv_B_F	2,5	3,57	2
hMpv_B_R	2,5	3,57	2
hBoca1_F	2,5	3,57	2
hBoca1_R	2,5	3,57	2
hBoca2_F	2,5	3,57	2
hBoca2_R	2,5	3,57	2
hBoca3_F	2,5	3,57	2
hBoca3_R	2,5	3,57	2
RSV_A_v6_F	2,5	3,57	2
RSV_A_v6_R	2,5	3,57	2

Primer name	Volume of 100 pM primer stock, µl	Primer concentration in pool, pM/µl	Final concentration of primers in the working pool, pM/µl
RSV_B_1023_F	2,5	3,57	2
RSV_B_1023_R	2,5	3,57	2

Table S2b. Composition of primer pool 2

Primer name	Volume of 100 pM primer stock, µl	Primer concentration in pool, pM/µl	Final concentration of primers in the working pool, pM/µl
Bvic_v5_F	2,5	3,57	2
Bvic_v5_R	2,5	3,57	2
InfA-N1-F	2,5	3,57	2
InfA-N1-R	2,5	3,57	2
InfA-N2-F	2,5	3,57	2
InfA-N2-R	2,5	3,57	2
HAdV-B_F	2,5	3,57	2
HAdV-B_R	2,5	3,57	2
HAdV-C_F	2,5	3,57	2
HAdV-C_R	2,5	3,57	2
HAdV-E_F	2,5	3,57	2
HAdV-E_R	2,5	3,57	2
NL63_v3_F	2,5	3,57	2
NL63_v3_R	2,5	3,57	2
229E_F	2,5	3,57	2
229E_R	2,5	3,57	2
OC43_F	2,5	3,57	2
OC43_R	2,5	3,57	2
HKU1_F	2,5	3,57	2
HKU1_R	2,5	3,57	2
SARS-CoV-2_G1_F	2,5	3,57	2
SARS-CoV-2_G1_R	2,5	3,57	2
VZV_F	2,5	3,57	2
VZV_R	2,5	3,57	2
CMV_ex2_F	2,5	3,57	2
CMV_ex2_R	2,5	3,57	2
EBV_F	2,5	3,57	2
EBV_R	2,5	3,57	2

Table S3. Composition of the control panels

Control panel name					
**Panel 1**	**Panel 2**	**Panel 3**	**Panel 4**	**Panel 5**	**Panel 6**
HBoV-1	VZV	HPIV-1	HMpV-A	FluA/H1N1	HCoV-229E
HAdV-B	CMV	HPIV-2	HMpV-B	FluA/H3N2	HCoV-OC43
HAdV-C	EBV	HPIV-3	HRSV-A	SARS-CoV-2	HCoV-HKU1
HAdV-E		HPIV-4a	HRSV-B	FluB/Victoria	HCoV-NL63

Table S4. Analytical sensitivity of mp-tNGS

Concentration copies/ml	Number of positive duplicates/total number of test duplicates for each dilution (positive rate, %)											
	HBoV-1	HAdV-B	HAdV-C	HAdV-E	VZV	CMV	EBV	HPIV-1	HPIV-2	HPIV-3	HPIV-4a	HMpV-A
1*10^5	4/4 (100%)	4/4 (100%)	4/4 (100%)	4/4 (100%)	4/4 (100%)	4/4 (100%)	4/4 (100%)	4/4 (100%)	4/4 (100%)	4/4 (100%)	4/4 (100%)	4/4 (100%)
5*10^4	4/4 (100%)	4/4 (100%)	4/4 (100%)	4/4 (100%)	4/4 (100%)	4/4 (100%)	4/4 (100%)	3/4 (75%)	4/4 (100%)	4/4 (100%)	4/4 (100%)	4/4 (100%)
1*10^4	4/4 (100%)	4/4 (100%)	4/4 (100%)	4/4 (100%)	4/4 (100%)	4/4 (100%)	4/4 (100%)	0/4 (0%)	4/4 (100%)	4/4 (100%)	4/4 (100%)	4/4 (100%)
5*10^3	4/4 (100%)	4/4 (100%)	4/4 (100%)	4/4 (100%)	4/4 (100%)	4/4 (100%)	4/4 (100%)	1/4 (25%)	4/4 (100%)	4/4 (100%)	4/4 (100%)	1/4 (25%)
1*10^3	4/4 (100%)	4/4 (100%)	4/4 (100%)	4/4 (100%)	4/4 (100%)	4/4 (100%)	4/4 (100%)	1/4 (25%)	3/4 (75%)	4/4 (100%)	3/4 (75%)	1/4 (25%)
0	0/4 (0%)	0/4 (0%)	0/4 (0%)	0/4 (0%)	0/4 (0%)	0/4 (0%)	0/4 (0%)	0/4 (0%)	0/4 (0%)	0/4 (0%)	0/4 (0%)	0/4 (0%)

	HMpV-B	HRSV-A	HRSV-B	FluA/H1N1	FluA/H3N2	SARS-CoV-2	FluB/Victoria	HCoV-229E	HCoV-OC43	HCoV-HKU1	HCoV-NL63
1*10^5	4/4 (100%)	4/4 (100%)	4/4 (100%)	4/4 (100%)	4/4 (100%)	4/4 (100%)	4/4 (100%)	4/4 (100%)	4/4 (100%)	4/4 (100%)	4/4 (100%)
5*10^4	3/4 (75%)	4/4 (100%)	4/4 (100%)	4/4 (100%)	4/4 (100%)	4/4 (100%)	4/4 (100%)	4/4 (100%)	4/4 (100%)	3/4 (75%)	4/4 (100%)
1*10^4	1/4 (25%)	4/4 (100%)	4/4 (100%)	4/4 (100%)	4/4 (100%)	4/4 (100%)	4/4 (100%)	4/4 (100%)	4/4 (100%)	0/4 (0%)	4/4 (100%)
5*10^3	1/4 (25%)	3/4 (75%)	2/4 (50%)	4/4 (100%)	4/4 (100%)	4/4 (100%)	3/4 (75%)	4/4 (100%)	3/4 (75%)	2/4 (50%)	2/4 (50%)
1*10^3	0/4 (0%)	2/4 (50%)	2/4 (50%)	4/4 (100%)	0/4 (0%)	4/4 (100%)	0/4 (0%)	2/4 (50%)	3/4 (75%)	0/4 (0%)	0/4 (0%)
0	0/4 (0%)	0/4 (0%)	0/4 (0%)	0/4 (0%)	0/4 (0%)	0/4 (0%)	0/4 (0%)	0/4 (0%)	0/4 (0%)	0/4 (0%)	0/4 (0%)
